# Overexpression of *OsMYBS1* affect leaf morphology, photosynthesis, and agronomic performance in rice

**DOI:** 10.3389/fpls.2025.1653514

**Published:** 2025-09-30

**Authors:** Yinhua Ma, Jing Zhang, Kai Liang, Anqi Huang, Jingshu Liu, Weiwei Li, Bin Sun, Yan Zhao

**Affiliations:** ^1^ College of Agriculture and Biotechnology, Hunan University of Humanities, Science and Technology, Loudi, China; ^2^ State Key Laboratory of Hybrid Rice, Hunan Hybrid Rice Research Center, Hunan Academy of Agricultural Sciences, Changsha, China

**Keywords:** rice, MYB-related transcription factors, leaf yellowing, plant growth and development, chloroplast

## Abstract

MYB transcription factors are critical regulators of plant growth, development, and responses to environmental stresses. However, the role of OsMYBS1, a MYB-related transcription factor in rice (*Oryza sativa*), in modulating plant morphology and agronomic traits remains largely unexplored. In this study, we investigated the biological function of *OsMYBS1* by generating overexpression lines in rice. Our results demonstrated that overexpression of *OsMYBS1* led to pronounced morphological and physiological alterations, including reduced plant height, broader and yellowing flag leaves, and decreased chlorophyll content. Agronomic evaluations further revealed that overexpression lines produced more tillers and panicles, with a reduced seed-setting rate but without a decrease in total grain yield per plant. Transcriptomic analysis identified a substantial number of differentially expressed genes (DEGs) associated with chloroplast biogenesis, photosynthesis, and metabolic processes, indicating that OsMYBS1 may influence chloroplast development and function. Collectively, these findings suggest that OsMYBS1 functions as a multifunctional regulator of plant growth and development, modulating leaf morphology and key agronomic traits. Our study provides a foundation for future investigations into the molecular mechanisms governed by OsMYBS1 and its potential utility in rice breeding to improve key agronomic traits.

## Introduction

Rice (*Oryza sativa*) is a staple crop that sustains over half of the world’s population (Bin Rahman and Zhang, 2023). Enhancing rice yield and quality has long been a key focus of agricultural research. Plant growth, development, and productivity are regulated by a complex network of genes, with transcription factors playing a critical role in these processes (Strader et al., 2022; Dhatterwal et al., 2024). Among these, MYB transcription factors constitute one of the largest and most diverse families of transcription factors in the plant kingdom. They are known to regulate various biological processes, including cell differentiation, secondary metabolism, and responses to abiotic and biotic stresses (Dubos et al., 2010; Li et al., 2019; Liu and Osbourn, 2015; Millard and Kragelund, 2019; Wang et al., 2021; Wu et al., 2022).

The MYB family can be classified into four subgroups based on the number of adjacent repeats in the MYB domain including 1R-MYB (or MYB-related), R2R3-MYB, 3R-MYB, and 4R-MYB (Millard and Kragelund, 2019; Wu et al., 2022). In rice, this family comprises 62 1R-MYB genes, 88 R2R3-MYB genes, 4 3R-MYB genes, and 1 4R-MYB gene, accounting for approximately 40%, 56.77%, 2.58%, and 0.64% of the total MYB genes, respectively (Katiyar et al., 2012). Rice MYB transcription factors have been reported to participate in a variety of developmental and physiological processes, such as plant architecture, leaf morphology, and responses to abiotic stresses and biotic stresses (Guo et al., 2023; Li et al., 2019; Liu et al., 2024; Xiong et al., 2014; Yang et al., 2012; Yang W. T. et al., 2014; Zhang et al., 2024). Among these, the R2R3-MYB genes have long been extensively studied due to their large size. For instance, the R2R3-type OsMYB30 is a versatile transcription factor in rice that regulates both biotic and abiotic stress responses, as well as developmental processes. OsMYB30 negatively regulates cold tolerance by interacting with JAZ proteins to inhibit β-amylase expression (Lv et al., 2017), improve pathogen resistance by enhances lignification and cell wall strengthening (Li et al., 2020), contributes to brown planthopper resistance by regulating phenylalanine ammonia-lyase (PAL) pathways (He et al., 2020) and confer aluminum resistance in acidic soils (Gao et al., 2023). Overexpression of R2R3-MYB gene *OsMYB2* improves rice tolerance to salt, cold, and dehydration stresses (Yang et al., 2012). The R2R3-MYB OsMYB1, OsMYB2P-1 and OsMYB4P have been reported to be involved in phosphate acquisition in rice (Dai et al., 2012; Gu et al., 2017; Yang C et al., 2014).

In contrast to the R2R3-MYB genes, the MYB-related genes have attracted much less attention, and only a few have been functionally studied. The MYB-related gene *OsMYB48–1* enhances drought and salt tolerance by regulating abscisic acid (ABA)-dependent signaling pathways (Xiong et al., 2014), while *OsMYB1R1* negatively regulates drought resistance, conferring improved drought tolerance and decreased ABA sensitivity in rice (Peng et al., 2023; Yin et al., 2017). Despite extensive research on the MYB gene family, the specific roles of many individual MYB genes in rice remain unclear, especially those of the MYB-related genes. *OsMYBS1* encodes a 1R-MYB protein that was first reported to mediate sugar and hormone regulation of α-amylase gene expression (Lu et al., 2002). It also reported to regulate defense-related gene expression (Li et al., 2017). However, its potential regulatory role in rice growth, morphogenesis, and productivity has not been thoroughly explored. Recently, MYB transcription factors MpMYB2/5 and AtMYBS1/AtMYBS2 have been reported to control chlorophyll biosynthesis and photosynthesis-associated gene expression (Frangedakis et al., 2024). Given that OsMYBS1 belongs to the same RR-MYB clade of 1R-MYB as MYB2 and MYB5, it is plausible that it also participates in chloroplast biogenesis and photosynthesis-related processes in rice. However, this potential function has not yet been investigated. Given that photosynthesis efficiency and plant architecture are critical factors influencing crop yield, understanding the role of *OsMYBS1* in these processes is essential.

In this study, we investigated the function of *OsMYBS1* by generating overexpression lines in rice. Our analyses focused on leaf morphology, chlorophyll content, and key agronomic traits at the maturity stage. Overexpression of *OsMYBS1* resulted in altered leaf morphology, reduced plant height, increased tiller and panicle numbers, and a decreased seed-setting rate compared to wild-type plants. These findings provide new insights into the role of *OsMYBS1* in rice growth and development, contributing to a broader understanding of MYB transcription factors. Our study paves the way for future research into the molecular mechanisms regulated by *OsMYBS1* and its potential applications in rice breeding to improve agronomic traits and resilience.

## Materials and methods

### Plant material

The overexpression transgenic lines were obtained on the *japonica* rice cultivar Hejiang19 background. Thus, Hejiang19 was used as the wild-type control for all morphological and molecular analyses. The template for gene amplification was obtained from the cDNA of the *indica* rice variety Kasalath. Experimental plants were grown in the field or in the greenhouse under standard management practices at the Genetics Institute of Wuhan University in Wuhan, China. To investigate the effect of overexpression of *OsMYBS1* on agronomic performance, the chlorophyll content and photosynthetic rate measurement, plants were grown in Wuhan, China (latitude 30834’N; longitude 114817’E), under a standard field management regime for the region as in the previous study (Zhao et al., 2016). Seedlings, 30-day-old, of all experimental materials were transplanted in the field in May, with 16.7-cm spacing between plants within each line and 26.7 cm between rows. For expression analysis and protoplast isolation, plants were planted under controlled environmental conditions (26 °C ± 0.5 °C, 16-h-light/8-h-dark cycle).

### Plasmid construction and rice transformation

To generate the overexpression construct (OE-*OsMYBS1*), a 921-bp cDNA fragment corresponding to the full-length *OsMYBS1* coding sequence was amplified via PCR using cDNA from the Kasalath rice variety as a template. The resulting DNA fragment was inserted into the pCXUN vector, which had been pre-digested with XcmI. The pCXUN vector features a maize ubiquitin promoter for driving gene expression and a *nos* terminator for transcriptional termination.

The constructs were introduced into *Agrobacterium tumefaciens* EHA105 via electroporation. The *Agrobacterium* mediated transformations of rice (Hejiang19) were carried out as previously described (Chen et al., 2007).

### Southern blot analysis

The procedure was carried out as previously described (Ma et al., 2017). Briefly, a probe (**Supplementary Table S1**) was labeled with [α-32^P^] dCTP using the Prime-a-Gene labeling system (Promega). Twenty micrograms of genomic DNA were digested with the EcoRI restriction enzyme (Fermentas), separated on a 1% agarose gel, and transferred to a Hybond-NC nylon membrane (Amersham Biosciences). The membrane was prehybridized at 65 °C for 3 hours, followed by hybridization with the labeled probe in fresh hybridization buffer for 12 hours at 65 °C. After hybridization, the membrane was washed at 65 °C for 15 minutes in 2× SSC containing 0.2% SDS, then for another 15 minutes in 1× SSC with 0.1% SDS. Finally, the hybridization signals were visualized using a Typhoon PhosphorImager (Amersham Biosciences) after exposing the membrane to storage phosphor screens.

### Quantitative real-time PCR

**Figure 1:**
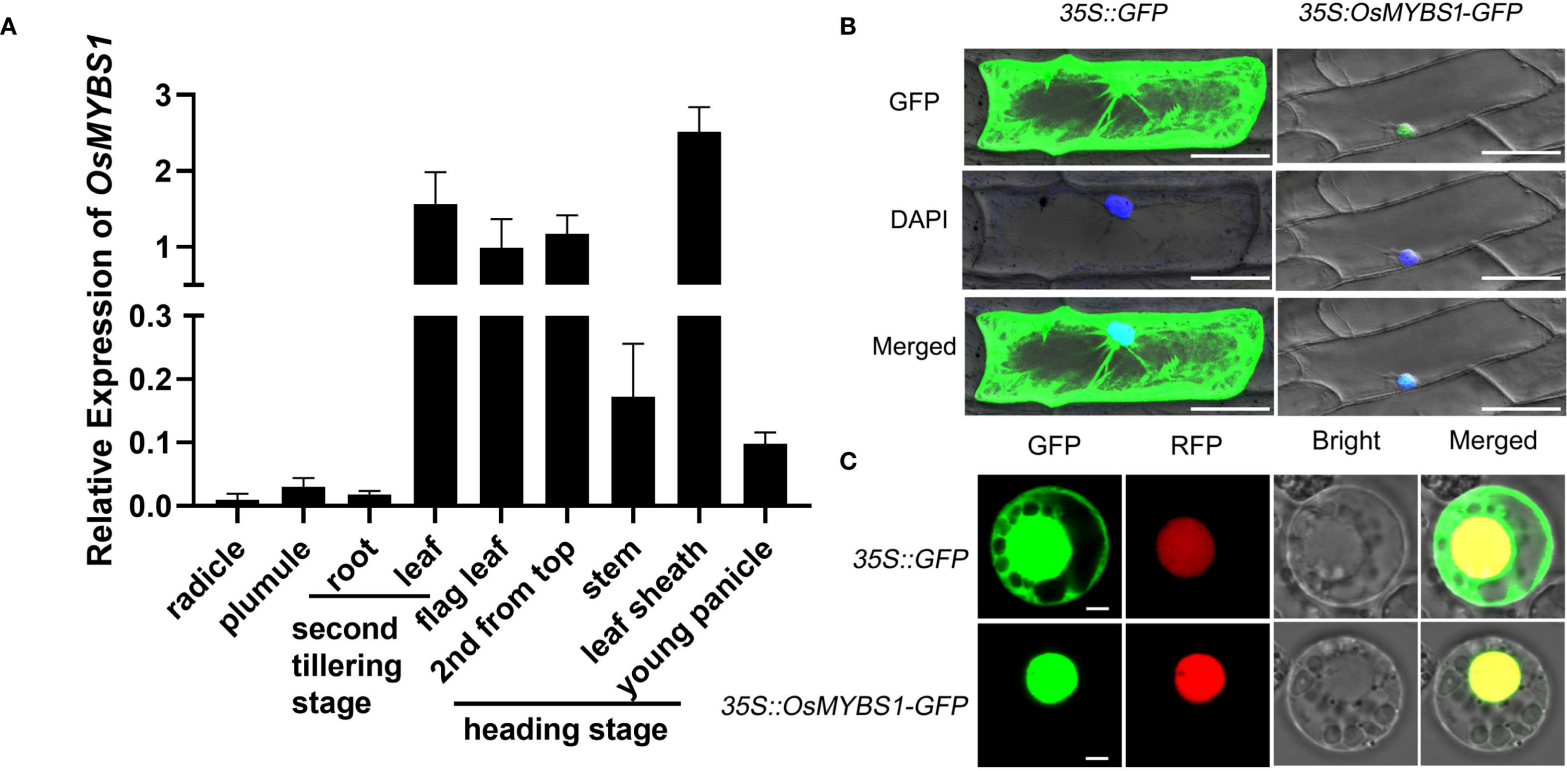
Expression and subcellular localization of OsMYBS1. **(A)** Expression of *OsMYBS1* in various organs determined by qRT-PCR analysis. Rice ACTIN1 gene was used as an internal control. Error bars represent the SD of transcript levels determined from three independent biological replicates each time with three technical replicates. **(B)** OsMYBS1 subcellular localization in onion epidermal cells. *35S::GFP* (left) and *35S:: OsMYBS1-GFP* fusion gene (right) were transiently expressed in onion epidermal cells. Nucleus were visualized by DAPI staining (blue), *Bars* = 100μm **(C)** Subcellular localization of *OsMYBS1* in rice protoplasts. GFP, fluorescence of GFP-*OsMYBS1* fusion protein. RFP, fluorescence of nuclear marker 63zip-RFP fusion protein. Bright, bright-field. Merged, merged image of GFP, RFP and Bright. *Bars* = 3μm. The subcellular localization assay was replicated three times with consistent results.

For expression analysis of **Figure 1A**, total RNA was extracted from various rice tissues, including radicle and plumule at 48 hours post-emergence, roots and leaves at the second tillering stage, as well as flag leaves, the second leaf from the top, stems, leaf sheaths, and young panicles at the heading stage. RNA extraction was performed using TRIzol reagent (TaKaRa) following the manufacturer’s protocol and then treated with DNase I (Fermentas) to eliminate genomic DNA contamination. The subsequent sample used for cDNA synthesis was carried out with the RevertAid™ First Strand cDNA Synthesis Kit (Fermentas), according to the manufacturer’s instructions. Quantitative PCR was performed on the synthesized cDNA using specific primers and SYBR Green PCR Master Mix (Applied Biosystems) in a CFX96 Real-Time System (Bio-Rad). The primers used for quantitative real time PCR are presented in **Supplementary Table S1**.

### Phylogenetic analysis of OsMYBS1

The protein sequence of OsMYBS1 was used to BLAST against the selected species in Phytozome (https://phytozome-next.jgi.doe.gov/). Sequences were then downloaded (**Supplementary Table S2**) and aligned with ClustalX Version 1.83 (Thompson et al., 1997). The generated FASTA sequences were then analyzed by MEGA11 (Tamura et al., 2021), using the the neighbor-joining (NJ) method. Bootstrap analyses of 1,000 replicates were carried out.

### Subcellular localization

For the particle bombardon of onion epidermal cells, the *OsMYBS1* coding sequence amplified by PCR was cloned into the downstream of the CaMV 35S promoter and in frame with GFP in the vector HBT95. The resulting plasmid DNA was bombarded into onion epidermal cells, using a helium biolistic device (Bio-Rad PDS-1000). Samples were examined by confocal laser-scanning microscopy (Olympus FV1000).

For rice protoplasts subcellular localization, the rice protoplasts were isolated from 10-day-old plants as previous described (Zhao et al., 2016). After transfection, the protoplasts were observed under an Olympus Fluoview FV1000 Laser Scanning Confocal Microscope. Nucleus marker was bZIP63 as described previously (Walter et al., 2004).

### RNA-sequencing and data analysis

For RNA sequencing, flag leaves from both Hejiang19 and the overexpression line OE-1 at the heading stage were collected. Each sample taking 5 leaves, and three biological replicates were included for each rice variety. Total RNA of each sample was extracted using TRIzol Reagent (Invitrogen). The samples were then sent to Suzhou Genewiz Corporation for transcriptional expression analysis. mRNA purification, cDNA preparation, end repair, adaptor ligation, and cDNA amplification were performed using the mRNA-Seq Sample Preparation Kit (Illumina). Libraries with different indices were multiplexed and loaded on an Illumina HiSeq instrument according to manufacturer’s instructions (Illumina, San Diego, CA, USA). Sequencing was carried out using a 2x150bp paired-end (PE) configuration; image analysis and base calling were conducted by the HiSeq Control Software (HCS) + OLB + GAPipeline-1.6 (Illumina) on the HiSeq instrument using an Illumina NovaSeq 6000 platform. Cutadapt (version 1.9.1) was used to pretreat the raw reads, filter low-quality data, and eliminate pollution and connector sequences. Raw reads were processed to remove adapter sequences and trimmed at the 5’ and 3’ ends to discard bases with Phred quality scores<20 or ambiguous nucleotides (N). Reads shorter than 75 bp after trimming were filtered out. The remaining high-quality clean reads were aligned to the *Oryza sativa* reference genome (MSU Rice Genome Annotation Project, Release 7, MSU v7.0) using Hisat2 (v2.0.1). Gene expression levels were quantified with HTSeq (v0.6.1) and normalized as fragments per kilobase of transcript per million mapped reads (FPKM). Differentially expressed genes (DEGs) were identified using the DESeq2 package (v1.6.3) in Bioconductor, with thresholds of |fold change| ≥ 2 and adjusted p-value (padj) ≤ 0.05. Gene Ontology (GO) and Kyoto Encyclopedia of Genes and Genomes (KEGG) enrichment analyses were conducted using the clusterProfiler package in Bioconductor. GO terms and KEGG pathways with a false discovery rate (FDR) ≤ 0.05 were considered significantly enriched.

### Chlorophyll content and photosynthetic rate measurement

When the rice plants reached the heading stage, three flag leaves were collected from each line. Leaves from both transgenic and WT plants (0.4 g each) were ground into powder in liquid nitrogen. A total of 0.2 g of the powdered sample was placed into a 10 mL centrifuge tube, followed by the addition of 5 mL of 80% acetone. The mixture was thoroughly mixed and incubated overnight at -20 °C in the dark, with periodic inversion to ensure proper mixing. The sample was then centrifuged at 10,000 g for 10 minutes, and the supernatant was collected. This process was repeated once, and the final supernatant was reserved for analysis. The absorbance of the supernatant was measured at 646 nm and 663 nm using a UV spectrophotometer (UV-1601, Shimadzu, Japan). The chlorophyll content in the leaves was calculated according to Lichtenthaler (1987) (Lichtenthaler, 1987) using the following equations:


$$\begin{array}{c} Ca=12.21A_{663}-2.59A_{646};Cb=20.13A_{646}-5.03A_{663};Ca+Cb\\ =17.54A_{646}+7.18A_{663} \end{array}$$

The total chlorophyll content (Ca + Cb) calculated from the formulas was subjected to statistical analysis.

The SPAD values of flag leaves at the heading stage was measured using the SPAD 502 Plus meter. The probe was positioned at the midpoint between the leaf midrib and the leaf edge (avoiding the midrib). Three readings were taken on both sides of the leaf, and the average of these six measurements was recorded as the SPAD value for each leaf. A total of 30 leaves were measured for each rice material, and the final SPAD value was calculated as the average of these measurements.

The photosynthetic rate of the flag leaves was measured for both OE-*OsMYBS1* and WT plants at the heading stage using a portable photosynthesis system (LI-6400, LI-COR). Measurements were performed under ambient CO_2_, temperature, and humidity, with saturating light intensity (1500 μmol m^−2^ s^−1^) supplied by the LI-6400 chamber. Measurements were taken from 4–6 leaves per plant between 10:00 AM and 1:00 PM.

### Paraffin cross-section assay

To analyze the detailed structure of the leaves, mature flag leaves, including at least the bottom half of the tissue, were used for a paraffin cross-section assay. The paraffin sectioning was performed by Wuhan Xavier Biotechnology Co., LTD. The sections were then observed using CaseViewer 2.0 software.

### Yield performance test in field

To investigate the effect of overexpression of *OsMYBS1* on agronomic performance, the plants were grown in Wuhan, China, under a standard field management regime for the region as in the previous study (Zhao et al., 2016). Seedlings, 30-day-old, of all experimental materials were transplanted in the field in May, with 16.7-cm spacing between plants within each line and 26.7 cm between rows. The plant height and the tiller number were examined at the heading stage. The panicle number, total grain number, filled grain number and the seed-setting rate were examined at harvest. The seed-setting rate was calculated as the percentage of full grains in all rice grains per plant. At least ten plants were randomly selected and examined.

## Results

### Characterization of *OsMYBS1* gene

**Figure 2:**
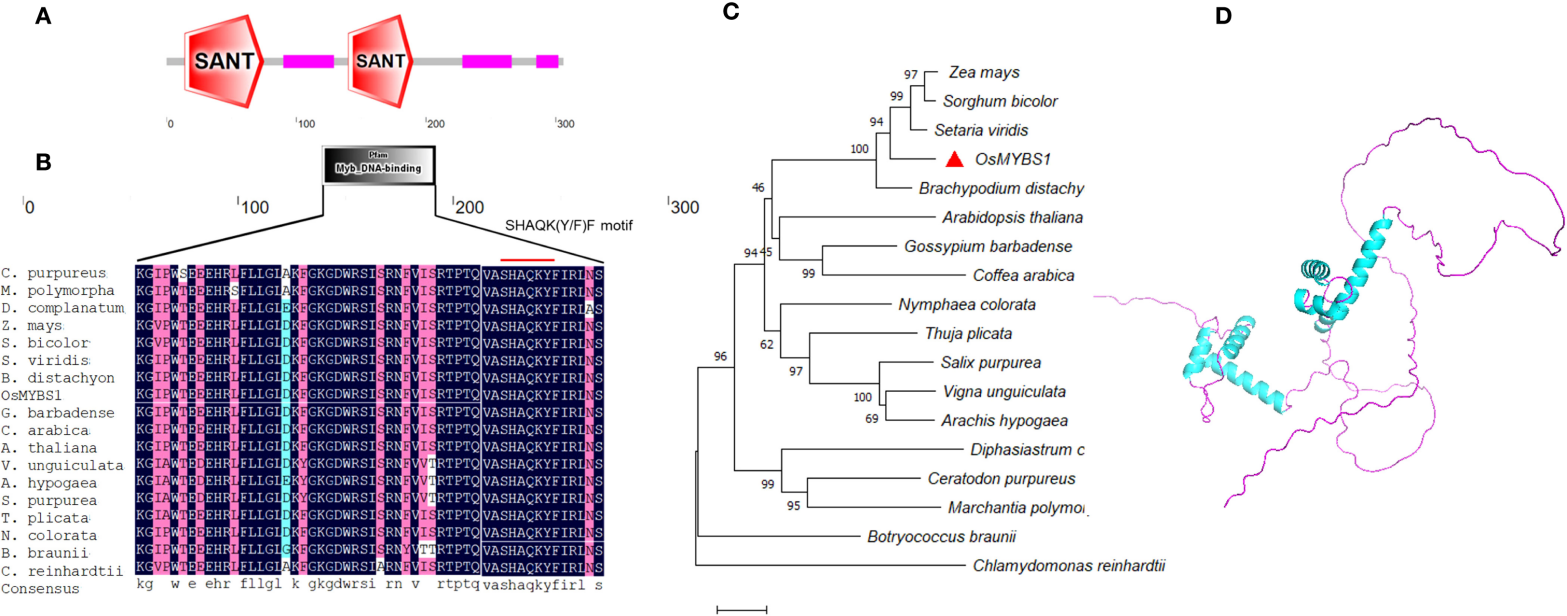
Sequence analysis of OsMYBS1. **(A)** Predicted domain of OsMYBS1 by SMART. **(B)** Alignment of the MYB domains of OsMYBS1 with OsMYBS1 like proteins in other representative species. The SHAQKYF motif which is conserved in 1RMYB proteins was indicated by the red line. Sequences are the same as in **(C)**. The accession IDs are as follows: *Zea mays*: RefGen_V4|Zm00001d040464_P001; *Sorghum bicolor*: v5.1|Sobic.003G132300.1.p; *Setaria viridis*: v4.1|Sevir.5G185000.1.p; *Brachypodium distachyon*: v2.1|Bradi2g13860.1.p; *Gossypium barbadense*: v1.1|Gobar.A09G050400.1.p; *Nymphaea colorata*: v1.2|Nycol.B00511.1.p; *Vigna unguiculata*: v1.1|Vigun02g014300.1.p; *Salix purpurea*: v5.1|Sapur.007G070800.5.p; *Coffea arabica*: v0.5|evm.model.Scaffold_578.301; *Arachis hypogaea*: v1.0|arahy.Tifrunner.gnm1.ann1.C8ILII.1; *Ceratodon purpureus*: v1.1|CepurGG1.4G124300.2.p; *Botryococcus braunii*: v2.1 |Bobra.341_2s0033.1.p; *Marchantia polymorpha*: v3.1|Mapoly0026s0070.2.p; *Arabidopsis thaliana*: AT1G49010; *Chlamydomonas reinhardtii*: v5.6|Cre10.g430750.t1.1; *Diphasiastrum complanatum*: v3.1|Dicom.Y128000.1.p; *Thuja plicata*: v3.1c|Thupl.29379524s0012.1.p. **(C)** Phylogenetic analysis of OsMYBS1 and other OsMYBS1 like proteins from various species. Sequences were obtained from Phytozome (https://phytozome-next.jgi.doe.gov/). Gene ID and sequences are listed in **Supplementary Table S1**. The evolutionary history was inferred using the Neighbor-Joining method. The optimal tree is shown. The percentage of replicate trees in which the associated taxa clustered together in the bootstrap test (1000 replicates) are shown next to the branches. The tree is drawn to scale, with branch lengths in the same units as those of the evolutionary distances used to infer the phylogenetic tree. **(D)** Predicted protein structure of OsMYBS1. The predicted structure was obtained from AlphaFold with the accession of Q8LH59 and then displayed by PyMOL (https://www.pymol.org/).

*OsMYBS1* was predicted to contain two SANT domains by the SMART tool (http://smart.embl.de/) (**Figure 2A**). The second SANT domain (amino acids 140-190) is also annotated as a Myb_DNA-binding domain (amino acids 143-188) (**Figure 2A**). This overlap aligns with the fact that the SANT domain was originally identified due to its homology with the DNA-binding domain of *c-myb* (Boyer et al., 2002). However, in contrast to 2R-MYB proteins, the two MYB repeats in OsMYBS1 protein are separated, with the second repeat being more closely related to the MYB domains. Based on this structural feature, OsMYBS1 belongs to the RR-MYB subgroup of the MYB-related proteins according to the previous report (Lu et al., 2002; Du et al., 2013). This subgroup contains the highly conserved SHAQK(Y/F)F motif within the Myb_DNA-binding domain (**Figure 2B**) (Du et al., 2013). It contains 302 amino acids, which is four amino acids fewer than the recorded MYBS1 sequence in the *Japonica* group (NP_001414962.1). The calculated molecular weight (Mw) of the deduced OsMYBS1 protein is 31.91 kDa, with a predicted isoelectric point of 7.90.

In order to analyze the genetic relationship between OsMYBS1 and its homologous proteins in other species, we performed a BLAST search of the amino acid sequence of OsMYBS1 against the Phytozome database (https://phytozome-next.jgi.doe.gov/). Representative species from different plant groups, including Phycophyta, Bryophyta, Pteridophyta, Gymnosperms, and Angiosperms were selected to construct a phylogenetic tree using MEGA11 with the Neighbor-Joining method (**Figures 2B, C**; **Supplementary Table S2**). The BLAST results show that species such as *Chlamydomonas reinhardtii* and *Botryococcus braunii* from the Chlorophytes group also contain genes homologous to *OsMYBS1*. Furthermore, the SHAQK(Y/F)F motif in the MYBS1-like genes from all these selected species is highly conserved (**Figure 2B**), indicating that *OsMYBS1* is an ancient and evolutionarily conserved gene across plant evolution.

Additionally, we obtained the predicted three-dimensional structure of OsMYBS1 from AlphaFold (https://alphafold.ebi.ac.uk/) (Varadi et al., 2024) with the accession number Q8LH59 (**Figure 2D**). The structure revealed that the non-MYB regions of OsMYBS1 contain extensive intrinsically disordered regions (IDRs). Notably, the non-MYB regions of OsMYBS1-like proteins show greater sequence diversity compared to the DNA-binding region, which may be important for the functional diversity of OsMYBS1 in different species. This variability in the non-MYB regions could contribute to the adaptation of OsMYBS1 to different functional roles across plant lineages.

### Expression and subcellular localization of OsMYBS1

To better characterize the function of *OsMYBS1* in rice, we examined the expression level of *OsMYBS1* across various rice organs and at different developmental stages by quantitative real-time PCR (qRT-PCR) (**Figure 1A**). These results demonstrated that *OsMYBS1* was highly expressed in leaf sheath at heading stage, as well as in leaves at different developmental stages. In contrast, its expression was low in radicle, plumule and root (**Figure 1A**). We also observed a relatively higher level of *OsMYBS1* expression in tiller buds compare to leaf at the initiation stage of tillering (**Supplementary Figure S1**). This expression pattern suggests that OsMYBS1 may play a more prominent role in the development of above-ground tissues, particularly during the heading stage.

To determine the subcellular localization of the OsMYBS1 protein, we constructed a fusion of the *OsMYBS1* coding region with a modified green fluorescent protein (GFP) at the N-terminus, driven by the CaMV 35S promoter. For the GFP channel, the fluorescence was observed in both the cytoplasm and the nucleus (**Figure 1B**, left panel). However, when the OsMYBS1-GFP fusion protein was introduced in onion epidermal cells via particle bombardment, fluorescence was exclusively detected in the nucleus, as confirmed by the blue DAPI staining (**Figure 1B**, right panel). A similar nuclear localization pattern was also observed when the construct was expressed in rice protoplasts. While GFP alone was distributed in both the nucleus and the cytoplasm, the OsMYBS1-GFP fusion protein was detected only in the nucleus, as verified by the nuclear marker 63zip-RFP (**Figure 1C**). These findings are consistent with the predicted role of OsMYBS1 as a transcription factor, suggesting that it functions in the nucleus to regulate gene expression.

### Overexpression of *OsMYBS1* affects leaf length and width

In order to elucidate the function of *OsMYBS1* in rice, we generated an *OsMYBS1* overexpression construct driven by the ubiquitin promoter and introduced into the *japonica* rice variety Hejiang19 (H1493) via *Agrobacterium. tumefaciens* mediated transformation (**Supplementary Figure S2**). Ten positive transgenic lines (OE-*OsMYBS1*) were successfully generated and confirmed by southern blot analysis. mong them, four lines (OE-1, OE-2, OE-3, and OE-4) contained a single copy (**Supplementary Figure S3A**). We chose three single copy lines (OE-1, 2 and 3) for further analysis. The expression level of *OsMYBS1* was confirmed by qRT-PCR, and all the three lines exhibited at least ten-fold increase in expression compared to WT plants (**Supplementary Figure S3B**).

**Figure 3:**
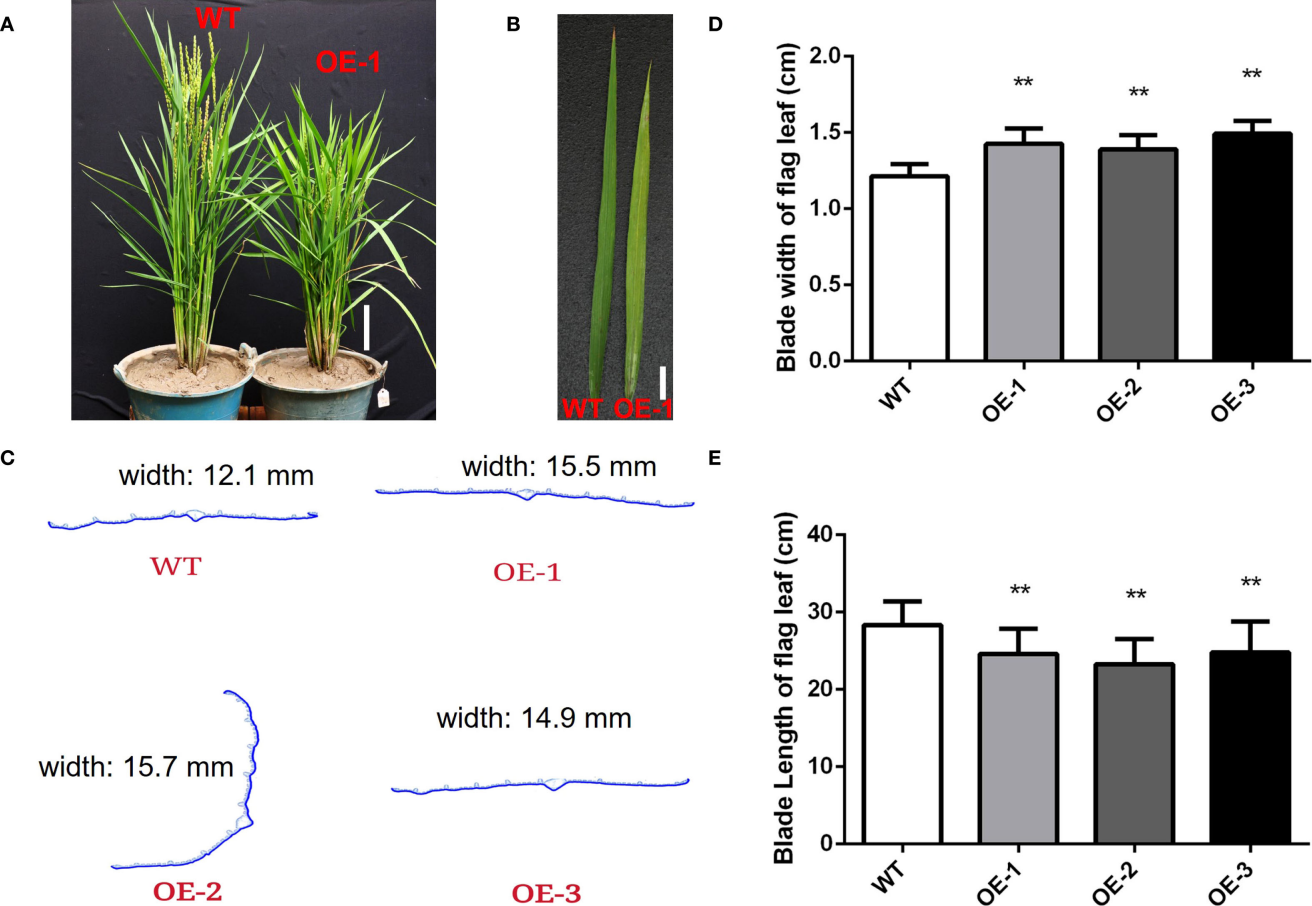
Overexpression of *OsMYBS1* affects leaf length and width. **(A)** The phenotype of the WT and OE-*OsMYBS1* plants at heading date. Bar= 10 cm. **(B)** The leaf phenotype of the WT and OE-*OsMYBS1* plants. Bar= 2 cm. **(C)** Paraffin section analysis on the flag leaves of the WT and OE-*OsMYBS1* plants. Samples were randomly selected during the heading date. **(D)** Statistic analysis of blade width of flag leaves. Flag leaf width was measured during the heading date. Data represent average value for at least 30 plants. Asterisks indicate a significant difference between WT plants and OE-*OsMYBS1* plants, **, *P*< 0.01. **(E)** Statistic analysis of blade length of flag leaves. Data represent average value for at least 30 plants. Asterisks indicate a significant difference between WT plants and OE-*OsMYBS1* plants according to a t-test, **, Student’s t-test, P< 0.01.

During the heading stage, the *OsMYBS1* overexpression lines showed significantly increased leaf width compared to the WT (**Figures 3A–D**). To further investigate this change, we randomly selected three leaves from each line and performed paraffin section analysis on the flag leaves at the heading stage. The average leaf width of WT plants was 12 mm, whereas the overexpression lines had an average width of nearly 15 mm (**Figures 3C, D**). Cross-section analysis revealed that there was no significant difference in cell size (**Supplementary Figures S4A, B**). In addition, both the number of large and small vascular bundles were increased in the overexpression line leaves (**Supplementary Figures S4B–D**). Thus, the increased leaf width in the overexpression lines is likely due to an increased number of cells. In contrast, the blade length of the flag leaves was significantly shorter in the overexpression transgenic lines compared to the WT plants (**Figure 3A, B, E**). These results suggest that *OsMYBS1* overexpression alters leaf morphology, resulting in wider but shorter flag leaves.

### Overexpression of *OsMYBS1* affects chlorophyll content and photosynthetic rate

**Figure 4:**
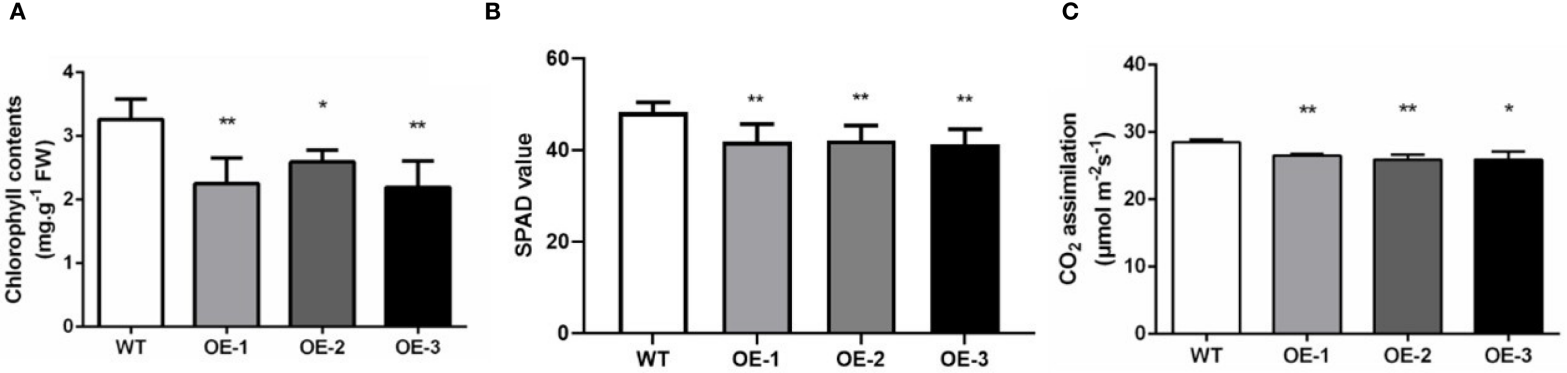
Chlorophyll content and photosynthetic rate of WT and OE-*OsMYBS1* plants. **(A)** Chlorophyll content of Chl a and Chl b total content in WT and OE-*OsMYBS1*flag leaves at the heading stage. **(B)** Flag leaf SPAD values at the heading stage. **(C)**The photosynthetic rate of flag leaves in WT and OE-*OsMYBS1* plants. Data represent average value for 30 plants. Asterisks indicate a significant difference between WT plants and OE-*OsMYBS1* plants according to a t-test, *, Student’s t-test, P< 0.05; **, Student’s t-test, P< 0.01.

During the heading stage, noticeable yellowing was observed in the leaves of the *OsMYBS1* overexpression transgenic lines whereas this phenotype was absent in WT plants (**Figures 3A, B**). Since leaf yellowing often is associated with a decline in chlorophyll content, we further assessed the chlorophyll content of chlorophyll a and chlorophyll b and total chlorophyll content. Both chlorophyll a and chlorophyll b contents were significantly reduced in the *OsMYBS1* overexpressing lines compared with WT plants, whereas the Chl a/b ratio showed no significant difference, indicating a coordinated reduction of pigments without altering photosystem antenna composition (**Figure 4A**, **Supplementary Figures S5A–C**).We also measured the SPAD (Soil and Plant Analyzer Development) values in the flag leaves using a SPAD-502 chlorophyll meter and the results further confirmed that chlorophyll content was significantly lower in the overexpressing lines (**Figure 4B**). Given that leaf chlorophyll content is a key determinant of photosynthesis rate, we next measured the photosynthetic rate in transgenic lines. The data showed that the photosynthetic rate of *OsMYBS1* overexpressing lines was significantly lower than that of the WT plants (**Figure 4C**). Consistent with the reduced photosynthetic capacity, the dry weight of overexpressing lines was significantly lower than that of WT (**Supplementary Figure S5D**), indicating impaired carbon assimilation and biomass accumulation.

### Agronomic traits of *OsMYBS1* overexpression transgenic plants

**Figure 5:**
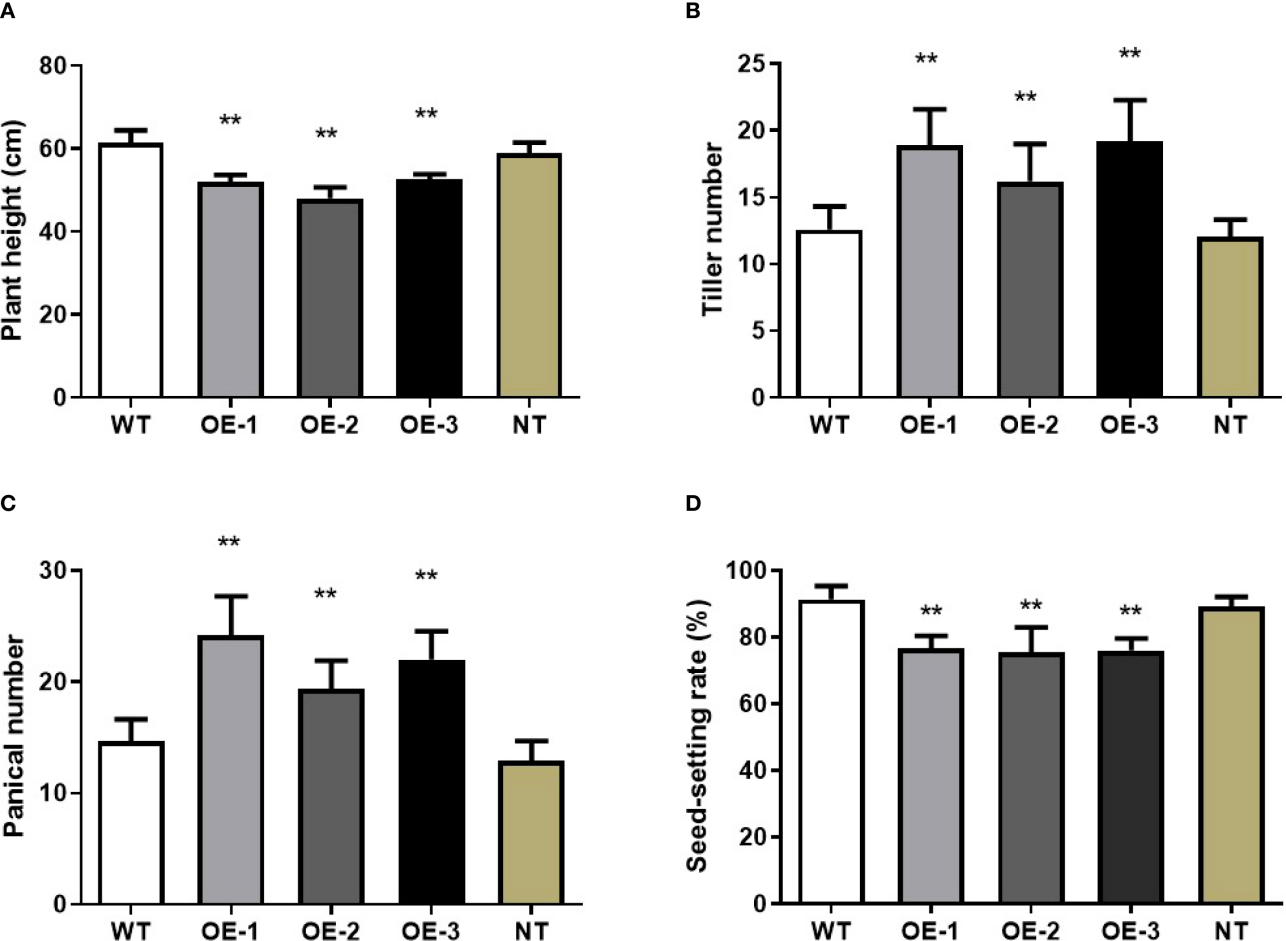
Pleiotropic effects of the *OsMYBS1* gene. **(A)** Plant height in WT plants and OE-*OsMYBS1* plants at the heading stage. **(B)** Tiller number of WT plants and OE-*OsMYBS1* plants at the tillering stage. **(C)** Panicle number of WT plants and OE-*OsMYBS1* plants. The above data represent average value for 20 plants. **(D)** The seed-setting rate of WT plants and OE-*OsMYBS1* plants. Seeds from 10 plants were measured after harvest. NT, negative transgenic. Asterisks indicate a significant difference at P< 0.01 between WT plants and OE-*OsMYBS1* plants according to a t-test.

In addition to alterations in leaf phenotype and photosynthetic rate, overexpression of *OsMYBS1* also affect other agronomic traits. Along with the reduction in leaf length, the height of the overexpression lines was notably shorter compared to the WT plants (**Figure 3A**). While the average height of the WT plants reached approximate 60 cm, the average plant height of OE lines was about 50 cm (**Figure 5A**). Furthermore, the heading date of the *OsMYBS1* overexpression transgenic plants occurred later than that of the WT (**Figure 3A**). The overexpressing lines also exhibited a significant increase in tiller number and panicle number (**Figures 5B, C**). However, despite these improvements in certain growth traits, the seed-setting rate was significantly reduced in the OE-*OsMYBS1* lines (**Figure 5D**). Although the seed setting rate is lower in OE-*OsMYBS1* plants, both the total grain number (**Supplementary Figure S6A**) and total filled grain number (**Supplementary Figure S6B**) were significantly higher in OE-1 and OE-3 compared with WT, whereas OE-2 displayed no significant difference but did not show a decreasing trend. Grain size, however, remained comparable between WT and OE lines. Taken together, these results indicate that yield per plant, as reflected by filled grain number, was either improved or at least maintained in the *OsMYBS1* overexpression lines. We also analyzed the phenotypes of the negative transgenic plants and found no significant differences compared to wild-type plants. These negative transgenic plants underwent the same transformation process but exhibited phenotypes similar to the wild-type, indicating that the transformation event itself had no effect on the observed phenotypic changes in this study.

### Overexpression of *OsMYBS1* significantly alter chloroplast-related gene expression

**Figure 6:**
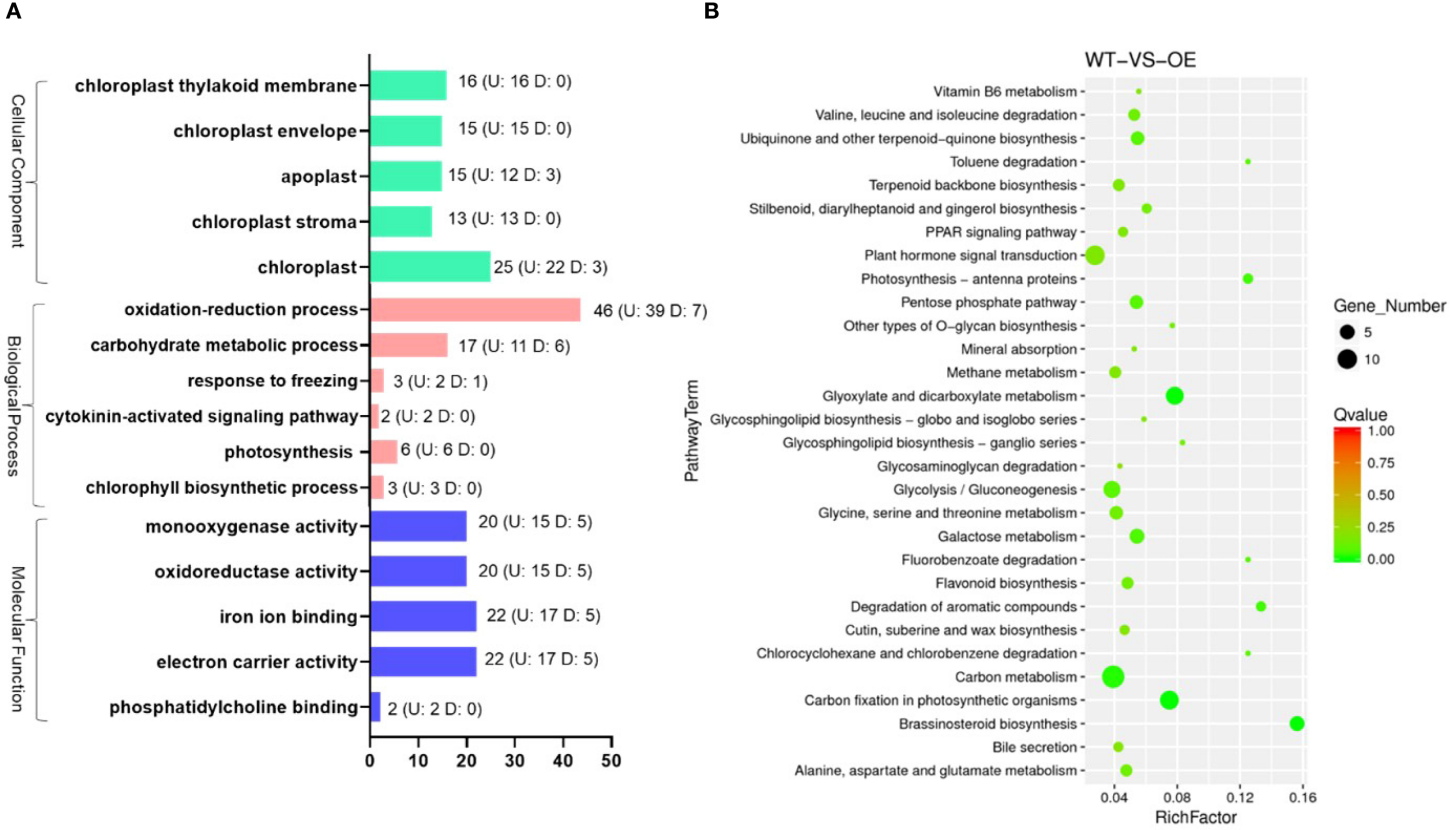
The GO and KEGG pathways containing the most abundant DEGs in OE-*OsMYBS1*. **(A)** The GO pathways containing the most abundant DEGs in OE-*OsMYBS1*. Numbers on the right of the bar indicate the up-regulated (U) or down-regulated **(D)** gene numbers in *OsMYBS1*overexpression plants categorized in this GO term. **(B)** The KEGG enrichment scatter plot for DEGs: the y-axis represents the pathway names, the x-axis represents the RichFactor, the size of the dots indicates the number of DEGs in each pathway, and the color of the dots corresponds to different Q-value ranges.

To identify the genes regulated by *OsMYBS1*, we performed transcriptome sequencing on the OE-1 line, as the morphological and physiological traits observed across all OE lines are similar. Differentially expressed genes (DEGs) were selected based on a minimum 2-fold change in FPKM values, resulting in a total of 523 genes, including 373 upregulated and 150 downregulated genes (**Supplementary Figure S7**). The DEGs from the WT and OE groups across the three biological replicates were clustered hierarchically in the heatmap. Most of the DEGs exhibited similar expression patterns across the three replicates, demonstrating stable upregulation or downregulation (**Supplementary Figure S7**). To categorize the functions of these DEGs, we analyzed their associated GO and KEGG pathways. The GO pathways with the highest number of DEGs are shown in **Figure 6A**. Among these GO pathways, chloroplast-related biological processes are particularly abundant (**Figure 6A**; **Supplementary Table S3**). These include processes related to chloroplast organelle components, such as chloroplast, chloroplast thylakoid membrane, chloroplast envelope, chloroplast stroma, as well as processes involving the chloroplast, such as oxidation-reduction process, electron carrier activity, carbohydrate metabolic process (**Figure 6A**). For example, the *OsAld-Y* (Os06g0608700) which participates in chlorophyll accumulation, chloroplast development and plant growth (Zhang et al., 2016), is significantly upregulated in OE-*OsMYBS1* plants (**Supplementary Table S3**). *Lhca4*/*DYE1* (Os08g0435900) encodes a subunit of the light-harvesting complex I (Yamatani H et al., 2018), is also up-regulated in OE-*OsMYBS1* plants (**Supplementary Table S3**). We manually examined the promoter regions (2 kb upstream of the ATG start codon) of the DEGs listed in **Supplementary Table S3**. Our analysis indicates that all but two of these genes contain at least one of the reported OsMYBS1 binding motif (GATAA). These results imply that *OsMYBS1* may also involve in chloroplast biogenesis by directly regulate these chloroplast-related genes. For KEGG analysis, the DEGs were found to be primarily enriched in plant hormone signal transduction, glyoxylate and dicarboxylate metabolism, glycolysis/gluconeogenesis, carbon metabolism, carbon fixation in photosynthetic organisms (**Figure 6B**). These results indicated that *OsMYBS1* may regulate leaf morphology and agronomic traits through modulating chloroplast-related genes expression and genes involved in hormone signaling.

## Discussion

In this study, we explored the functional role of the *OsMYBS1* gene in rice growth and development by analyzing overexpression lines of *OsMYBS1*. Our results demonstrated that *OsMYBS1* plays a significant role in regulating key agronomic traits, including plant height, leaf development, chlorophyll content, and overall productivity. These findings suggest that OsMYBS1 functions as an important regulator in rice morphogenesis and productivity.

Overexpression of *OsMYBS1* significantly changes leaf morphology, resulting in shorter and wider flag leaves compared to wild-type plants (**Figure 3**). Paraffin sectioning showed that the wider leaves of the overexpression lines mainly resulted from increased cell number rather than cell size, accompanied by a higher abundance of large and small vascular bundles. suggesting that OsMYBS1 promotes cell proliferation in leaves while potentially restricting stem elongation or modifying hormonal regulation. Consistent with this notion, cytokinin signaling was among the GO categories most enriched in differentially expressed genes in OE-*OsMYBS1* plants (**Figure 6**), implicating OsMYBS1 in hormonal crosstalk. Alternatively, enhanced leaf cell proliferation may lead to a trade-off in resource allocation, thereby limiting stem growth. Although net CO_2_ assimilation was significantly reduced in the overexpression lines, the decrease was relatively modest compared with their pronounced morphological alterations, indicating that impaired photosynthesis alone does not fully explain the observed growth defects. OsMYBS1 likely exerts a multifaceted regulatory role, coordinating cell proliferation, vascular differentiation, and hormone signaling. The precise mechanisms through which OsMYBS1 modulates cytokinin and other hormone signaling pathways remain to be fully elucidated. This is also consistent with findings on other MYB family genes, which often act as regulators of growth by modulating the expression of genes involved in hormone signaling pathways (Kim et al., 2024; Schmidt et al., 2013).

Overexpression of *OsMYBS1* markedly influenced yield-related traits by increasing tiller and panicle numbers while reducing the seed-setting rate, suggesting a trade-off between reproductive organ proliferation and fertilization efficiency. Despite this reduction, overall yield potential was preserved or even enhanced: OE-1 and OE-3 produced significantly more total and filled grains per plant, whereas OE-2 remained comparable to WT. Grain size was unaffected across all genotypes. These findings indicate that *OsMYBS1*-mediated changes in plant architecture and resource allocation can improve yield components without compromising overall productivity. To fully harness the beneficial trait of OsMYBS1, a more targeted expression strategy could be considered. For example, using a tiller bud-specific promoter to drive *OsMYBS1* expression could enhance yield potential while minimizing undesired phenotypic consequences, providing a promising approach for future improvement of rice productivity. We also observed noticeable yellowing in the leaves of *OsMYBS1* overexpression lines (**Figures 3A, B**). The yellowing of leaves in these transgenic lines indicates a possible disruption in chlorophyll biosynthesis or accelerated chlorophyll degradation. This observation is supported by the decreased chlorophyll content, implying that *OsMYBS1* may influence photosynthetic efficiency. Transcriptomic data further supports this, revealing that *OsMYBS1* overexpression significantly affects the expression of genes involved in chloroplast development and function (**Figure 6A**; **Supplementary Table S3**). In a recent study, MYB-related transcription factors Mp-RRMYB2/5 and AtMYBS1/2 were shown to regulate chloroplast biogenesis (Frangedakis et al., 2024). Since OsMYBS1 belongs to the same RR-MYB subgroup with Mp-RRMYB2/5 and AtMYBS1/2 (Frangedakis et al., 2024), it is plausible that it plays a similar role in chloroplast biogenesis in rice. This is supported by the upregulation of chloroplast-related gene expression in OE-*OsMYBS1* transgenic plants (**Figure 6A**; **Supplementary Table S3**). Moreover, this finding aligns with a previous report showing that MYBS1-binding is enriched in photosynthetic promoters at GATAA with MYBS1 binding to more than 50 photosynthetic genes (Halpape et al., 2023). Our results show that all but two of the DEGs listed in **Supplementary Table S3** contain at least one of the reported OsMYBS1 binding motif. Therefore, the yellowing phenotype observed in *OsMYBS1*-overexpressing plants could result from impaired chloroplast function, leading to reduced chlorophyll accumulation and compromised photosynthetic machinery, as the same phenotype in the *OsAld-Y/ygdl-1* mutant (Zhang et al., 2016). Furthermore, disruptions in chloroplast biogenesis may also accelerate cellular aging and senescence, providing a possible explanation for the premature aging phenotype observed in these plants. Although RR-MYB transcription factors were reported to positively regulate chloroplast biogenesis (Frangedakis et al., 2024), our study found that, *OsMYBS1* overexpression instead led to chloroplast-related dysfunction, resembling the phenotypic defects in mutant plants. There are several potential explanations for this unexpected outcome. Firstly, overexpression of *OsMYBS1* might lead to an imbalance in regulatory networks, causing misregulation of chloroplast-related genes. While normal levels of *OsMYBS1* may support proper chloroplast development, excessive expression could disrupt the fine-tuned coordination required for chloroplast biogenesis and maintenance. Secondly, overexpression might drive *OsMYBS1* expression in tissues or developmental stages where it is not normally active, leading to aberrant regulation of downstream targets. For example, genes critical for chloroplast formation might be misregulated in terms of timing or location, resulting in functional defects. Furthermore, high levels of *OsMYBS1* might trigger negative feedback loops, repressing essential components of chloroplast development. Alternatively, the overexpression might induce secondary effects, such as activating stress responses or senescence pathways, which impair chloroplast function. Consistent with the observed yellowing phenotype, *OsMYBS1* overexpression significantly decreased both Chl a and Chl b contents, while the Chl a/b ratio remained unchanged (**Supplementary Figure S5**). This indicates that the structural organization of photosystems is largely preserved, despite the overall reduction in chlorophyll pools. The concomitant reduction in dry weight further supports impaired carbon assimilation. Together with the enrichment of chloroplast-related DEGs revealed by transcriptome analysis, these results strongly suggest that OsMYBS1 plays a regulatory role in chloroplast function and photosynthetic capacity. However, further analyses, such as ultrastructural observations or quantification of chloroplast number, are needed to directly confirm the effect on chloroplast biogenesis. Taken together, these findings underscore the role of *OsMYBS1* as a potential regulator of chloroplast development and maintenance, impacting overall plant growth and physiological performance.

Overall, our findings suggest that *OsMYBS1* functions as a key regulator of rice growth and development, impacting both vegetative and reproductive traits. While its overexpression confers certain advantages, such as increased tillering, it also leads to trade-offs in plant height, leaf morphology, and seed-setting rate. Future studies should aim to elucidate the specific molecular pathways through which *OsMYBS1* modulates these traits and explore the potential of manipulating its expression for improving rice yield and stress tolerance.

## Data Availability

The datasets presented in this study can be found in online repositories. The names of the repository/repositories and accession number(s) can be found in the article/**Supplementary Material**.
