# Supplementary material for: Overexpression of OsMYBS1 affect leaf morphology, photosynthesis, and agronomic performance in rice
Source: Front Plant Sci. 2025 Sep 30;16:1653514. doi: 10.3389/fpls.2025.1653514 (PMC12518331; doi:10.3389/fpls.2025.1653514)
Supplement: Supplementary file 4 [file Supplementaryfile1.pdf]

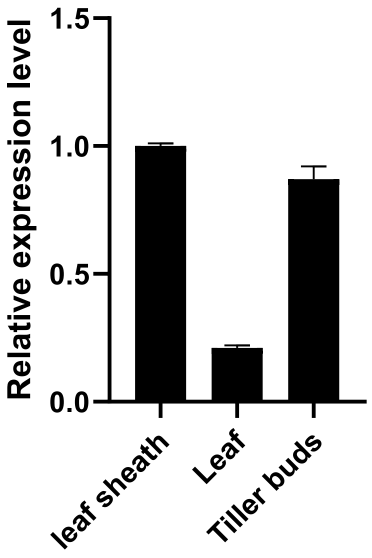

Figure S1 Expression of *OsMYBS1* in various organs at the initiation stage of tillering determined by qRT-PCR analysis. Rice ACTIN1 gene was used as an internal control. Error bars represent the SD of transcript levels determined from three independent replicates.

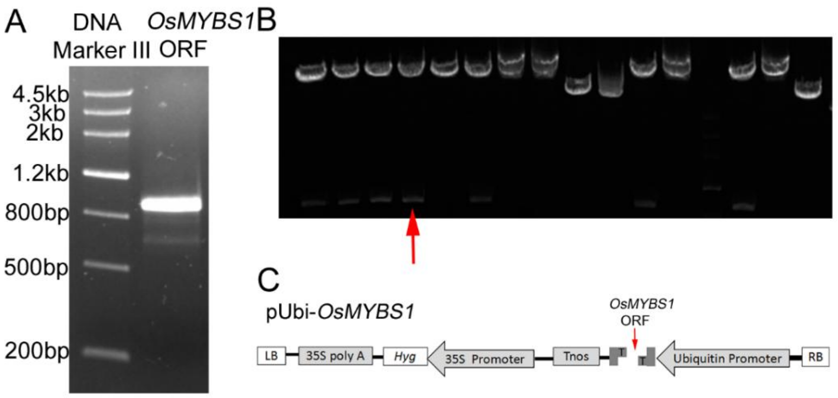

Figure S2 Construction of *OsMYBS1* overexpression vector. (A) Amplification of full - length ORF of *OsMYBS1* gene. (B) *OsMYBS1* full length ORF was ligated with the genetic transformation vector pCXUN and identified by recombinant vector BamHI digestion. (C) Construction of successful OE- *OsMYBS1*vector.

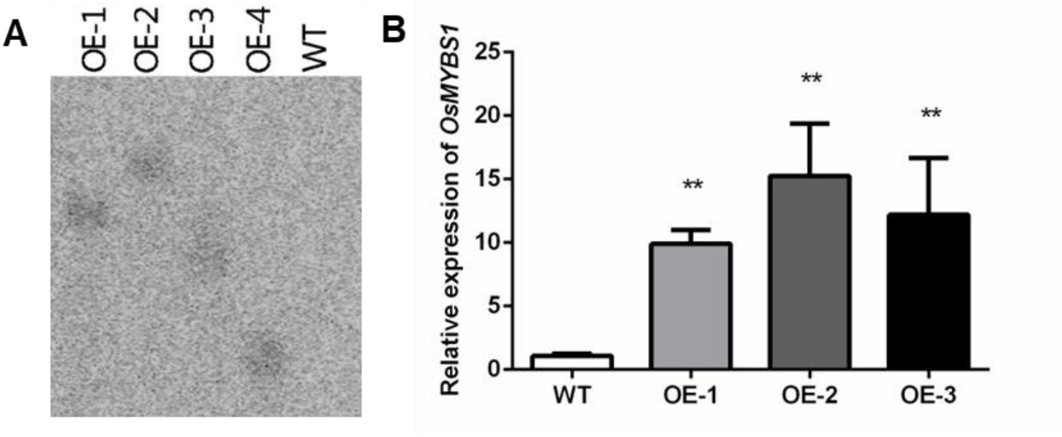

Figure S3 Southern blot analysis and expression confirmation of OE-*OsMYBS1* lines. (A) Southern blot analysis of OE-*OsMYBS1* transgenic plants. OE-1, OE-2, OE-3 and OE-4 were single copies of *OsMYBS* plants. (B) The relative expression levels of *OsMYBS1* in the WT and OE-*OsMYBS1* plants. Flag leaves at the heading stage were used in this experiment. Values are the means ± SD of three biological replicates.

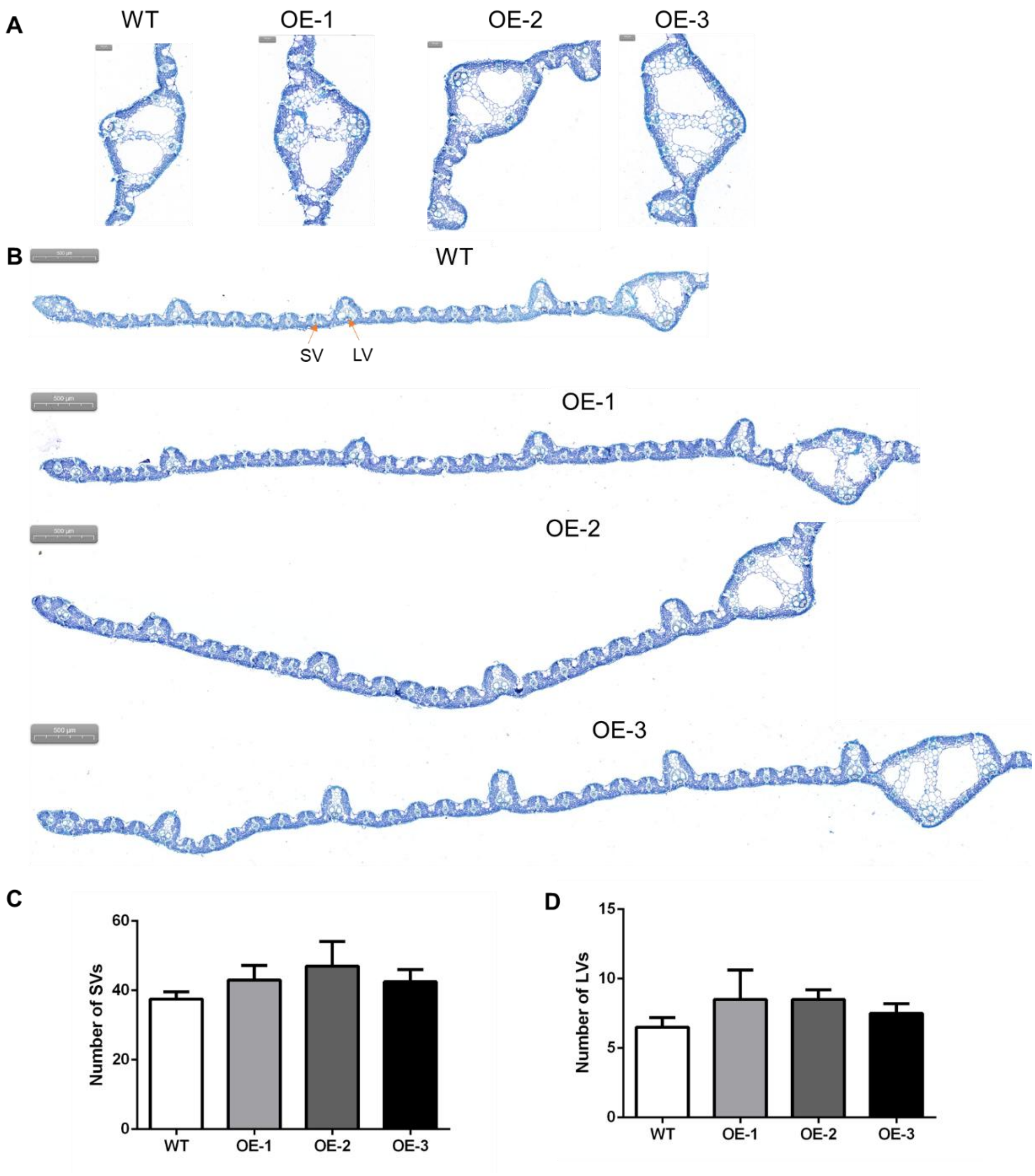

Figure S4 Paraffin section analysis of flag leaf cross sections in WT and OE-*OsMYBS1* plants. (A) Cross sections of flag leaves in WT and OE-*OsMYBS1* plants. Bars = 100 μm. (B) Transverse sections of the half expanded leaves of WT and OE-*OsMYBS1* plants. Bars = 500 μm. (C-D). Comparison of the number of small vascular bundles (SVs) (C) and large vascular bundles (LVs) (D).

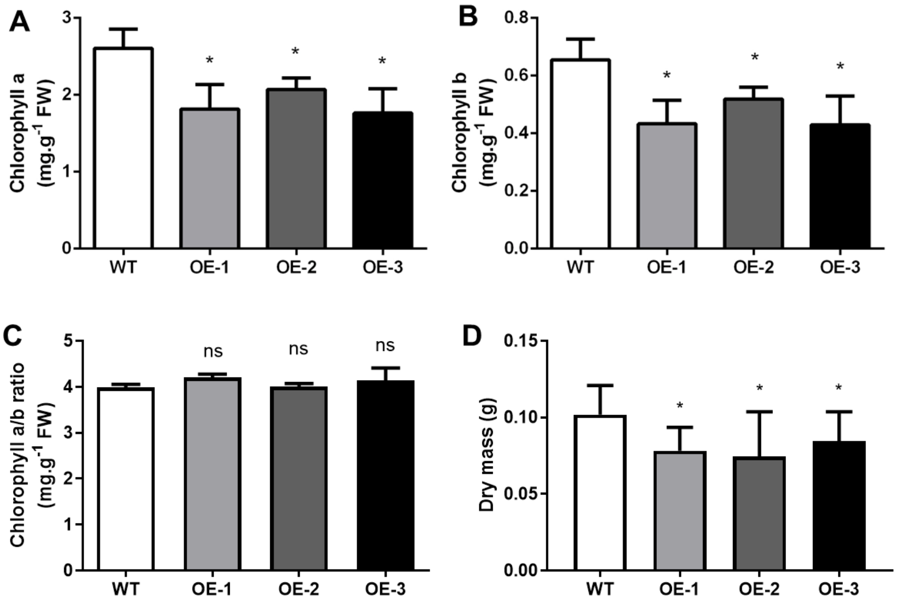

Figure S5 Chlorophyll content and dry mass in WT and OE-*OsMYBS1* plants. (A-B) Chlorophyll a and Chlorophyll b content in WT and OE-*OsMYBS1* plants. (C) The ratio of Chlorophyll a/b in WT and OE-*OsMYBS1* plants. (D) Dry mass of the WT and OE-*OsMYBS1* flag leaves at the heading stage. Ten flag leaves of the WT and OE-*OsMYBS1* plants were collected and measured. Asterisks indicate a significant difference between WT plants and OE-*OsMYBS1* plants according to a t-test, *, Student' s t-test, $P < 0.05$; ns, not significant.

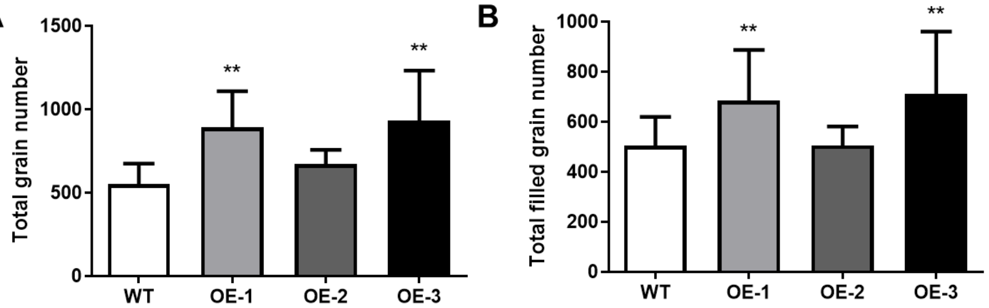

Figure S6 Total grain number and total filled grain number in WT and OE-OsMYBS1 plants. The above data represent average value for 10 plants. Asterisks indicate a significant difference at $P < 0.01$ between WT plants and OE-*OsMYBS1* plants according to a t-test..

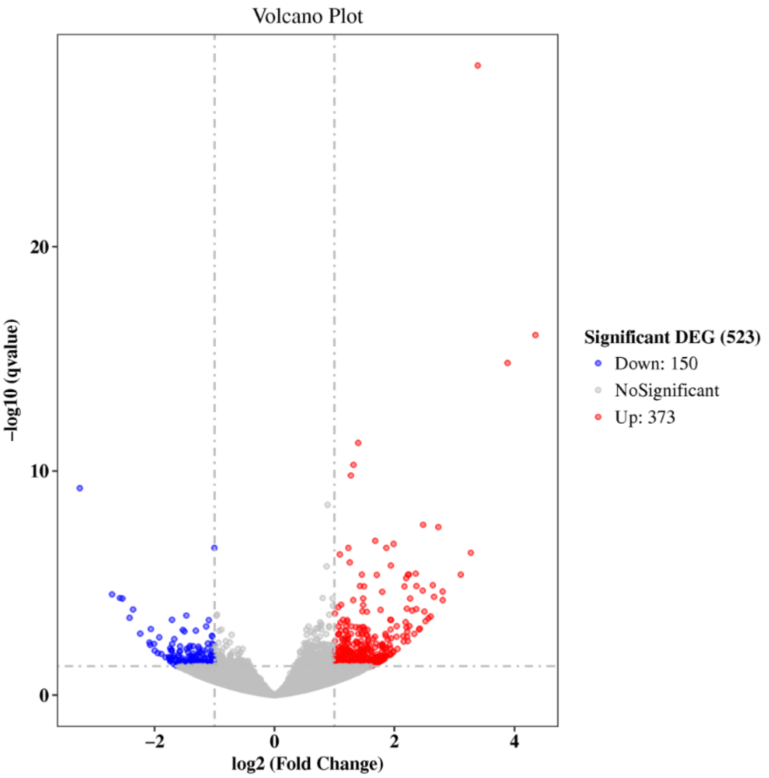

Figure S7 The volcano plot of DEGs. Red dots represent upregulated genes, while blue dots represent downregulated genes. The x-axis represents the fold change in gene expression across different samples, and the y-axis represents the statistical significance of the difference in gene expression.

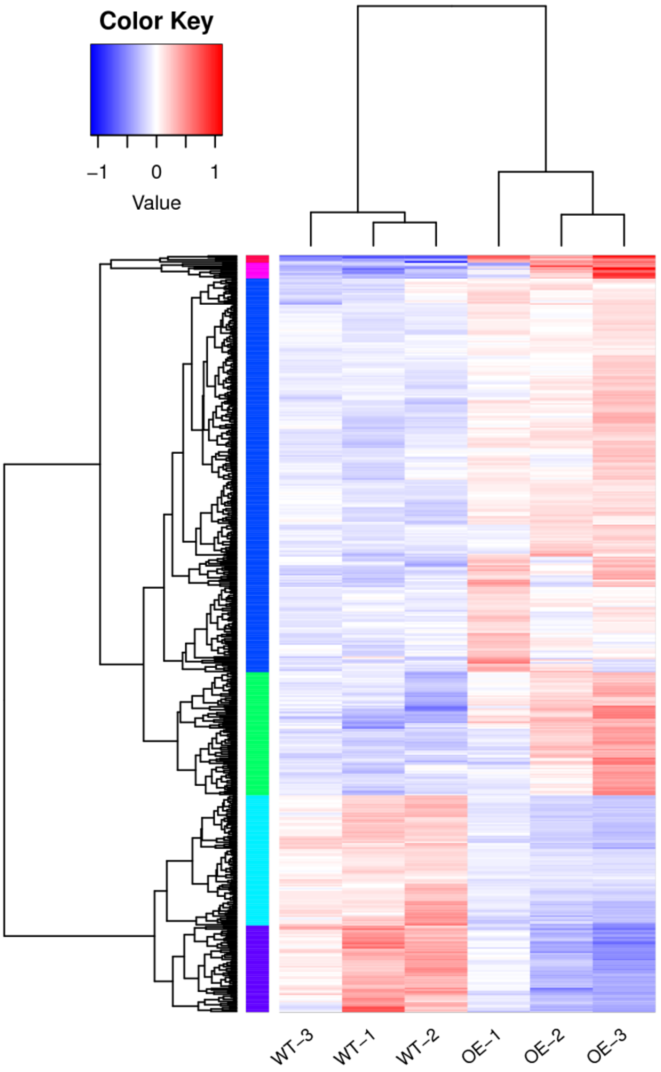

Figure S8 Hierarchical clustering analysis of DEGs based on log ratio of FPKM data. The color key represents FPKM normalized log2 transformed counts. Red represents up-regulated DEGs, green represents down-regulated DEGs. Each column represents a comparison group, each row represents a gene.
